# Re-Emergence of the Apicomplexan *Theileria equi* in the United States: Elimination of Persistent Infection and Transmission Risk

**DOI:** 10.1371/journal.pone.0044713

**Published:** 2012-09-06

**Authors:** Massaro W. Ueti, Robert H. Mealey, Lowell S. Kappmeyer, Stephen N. White, Nancy Kumpula-McWhirter, Angela M. Pelzel, Juanita F. Grause, Thomas O. Bunn, Andy Schwartz, Josie L. Traub-Dargatz, Amy Hendrickson, Benjamin Espy, Alan J. Guthrie, W. Kent Fowler, Donald P. Knowles

**Affiliations:** 1 Animal Diseases Research Unit, Agricultural Research Service, United States Department of Agriculture, Pullman, Washington, United States of America; 2 Department of Veterinary Microbiology and Pathology, Washington State University, Pullman, Washington, United States of America; 3 United States Department of Agriculture, Animal and Plant Health Inspection Service, Veterinary Services, Western Regional Office, Fort Collins, Colorado, United States of America; 4 Animal and Plant Health Inspection Service, Veterinary Services, National Veterinary Services Laboratories, Ames, Iowa, United States of America; 5 Texas Animal Health Commission, Austin, Texas, United States of America; 6 Department of Clinical Sciences, Animal Population Health Institute, Colorado State University, Fort Collins, Colorado, United States of America; 7 National Association of State Departments of Agriculture, Washington, District of Columbia, United States of America; 8 Equine Reproduction, San Antonio, Texas, United States of America; 9 Equine Research Centre, Faculty of Veterinary Science, University of Pretoria, Onderstepoort, South Africa; 10 California Department of Food and Agriculture, Sacramento, California, United States of America; University of Minnesota, United States of America

## Abstract

Arthropod-borne apicomplexan pathogens that cause asymptomatic persistent infections present a significant challenge due to their life-long transmission potential. Although anti-microbials have been used to ameliorate acute disease in animals and humans, chemotherapeutic efficacy for apicomplexan pathogen elimination from a persistently infected host and removal of transmission risk is largely unconfirmed. The recent re-emergence of the apicomplexan *Theileria equi* in U.S. horses prompted testing whether imidocarb dipropionate was able to eliminate *T. equi* from naturally infected horses and remove transmission risk. Following imidocarb treatment, levels of *T. equi* declined from a mean of 10^4.9^ organisms/ml of blood to undetectable by nested PCR in 24 of 25 naturally infected horses. Further, blood transfer from treated horses that became nested PCR negative failed to transmit to naïve splenectomized horses. Although these results were consistent with elimination of infection in 24 of 25 horses, *T. equi*-specific antibodies persisted in the majority of imidocarb treated horses. Imidocarb treatment was unsuccessful in one horse which remained infected as measured by nested PCR and retained the ability to infect a naïve recipient via intravenous blood transfer. However, a second round of treatment eliminated *T. equi* infection. These results support the utility of imidocarb chemotherapy for assistance in the control and eradication of this tick-borne pathogen. Successful imidocarb dipropionate treatment of persistently infected horses provides a tool to aid the global equine industry by removing transmission risk associated with infection and facilitating international movement of equids between endemic and non-endemic regions.

## Introduction

Effective strategies to control and eradicate arthropod-borne infectious diseases in animals and humans remain elusive [1]–[4]. Although large-scale immunization campaigns and/or arthropod control programs can be successful in preventing disease or the transmission of some vector-borne infections, diversity among pathogen strains and the presence of competent vectors can contribute to disease re-emergence [3], [5]–[8]. Arthropod-borne pathogens that establish persistent infections in their mammalian hosts represent a particularly challenging control problem. A broad range of pathogens including viruses, bacteria, and protozoan parasites can establish persistent infection without causing overt signs of disease [9]–[12]. Among the apicomplexan protozoan parasites, *Theileria equi*, closely related to bovine pathogens such as *Babesia bovis* and other *Theileria* spp, exemplifies this disease pattern [11], [13]–[15]. This tick-borne pathogen of horses can cause severe acute disease characterized by fever, anemia, hemoglobinuria and in some cases death. Infected horses that recover from acute infection become persistently infected for life with parasite loads ranging from 10^3^ to 10^6^ *T. equi*/ml of blood [14], [16], [17]. These persistently infected horses serve as sources of iatrogenic and tick-borne transmission [14], [17]. Iatrogenic blood transfer that transmitted *T. equi*, resulted in outbreaks of equine piroplasmosis associated with unsanctioned horse racing in Florida in 2008 [18]. In 2009, an outbreak of equine piroplasmosis in Texas was associated with natural transmission by *Amblyomma cajennense* and *Dermacentor variabilis* ticks, both of which are known biological vectors for *T. equi* [19], [20].

Although *T. equi* continues to be considered a foreign animal pathogen in the United States, the presence of infected horses, along with the discovery of ticks capable of transmitting parasites, raise the concern that *T. equi* could become widespread. Currently in the U.S., infected horses are managed under life-time quarantine with intensive transmission mitigation strategies. In isolated cases, euthanasia of infected horses has been used to control small outbreaks associated with limited iatrogenic transmission such as those in Florida [18]. However, euthanasia alone would be an inappropriate control strategy for outbreaks associated with natural vector transmission involving large numbers of horses as occurred in Texas. In addition to animal welfare concerns, attempting to control transmission by euthanasia of all *T. equi* infected horses would be costly due to the loss of valuable genetics, the economic value of individual animals, and expenses associated with long-term surveillance to monitor regional and national infection status. An effective control and eradication strategy that does not involve euthanasia is therefore desirable.

The purpose of the current study was to evaluate a chemotherapeutic approach for elimination of *T. equi* in naturally infected horses, with the goal of eliminating transmission risk. Several anti-protozoal drugs have been used with variable results for the treatment of *T. equi*, including imidocarb dipropionate, parvaquone, buparvaquone, and artemisinin derivatives [21]–[24]. However, the lack of a confirmatory test for parasite elimination and the variability in anti-protozoal drug susceptibility among different strains have contributed to the difficulty in determining chemotherapeutic efficacy [24]–[26]. Although our previous work demonstrated that imidocarb dipropionate eliminates *Babesia caballi* infection (another related vector-borne apicomplexan hemoprotozoan of horses) and removes the risk of experimental transmission by both iatrogenic blood transfer and tick feeding [26], the efficacy of imidocarb dipropionate for *T. equi* elimination in naturally, persistently infected horses is unknown. Imidocarb dipropionate (Imizol®; Schering Plough Animal Health) is an aromatic diaminide of the carbanilide series of antiprotozoal compounds, labeled for the treatment of babesiosis in dogs. Although the precise mechanism of action is not known, chromatin clumping, nuclear membrane dissolution, and cytoplasmic vacuolization of intraerythrocytic protozoan parasites occurs within 48 hours of imidocarb dipropionate treatment of infected horses [27]. Four intramuscular doses of 4 mg/kg of the dihydrochloride derivative of imidocarb, administered every 72 hours, reportedly eliminated *T. equi* infection in horses [21]. In contrast, the same dosing regimen of imidocarb dipropionate in naturally infected horses has been ineffective for *T. equi* elimination [22]. Moreover, imidocarb dipropionate treatment has resulted in transient lack of *T. equi* detection followed by recrudescence of parasitemia [24].

Based on studies to date, the efficacy of imidocarb dipropionate for the elimination of *T. equi* infection remains unclear. In the study reported herein, we tested whether imidocarb dipropionate eliminates *T. equi* infection from naturally infected horses and removes transmission risk. Parasite elimination from naturally infected horses was determined by quantitative PCR and nested PCR at multiple time-points following imidocarb treatment, and post-treatment transmission risk was assessed by blood transfer into susceptible naïve splenectomized horses.

**Figure 1:**
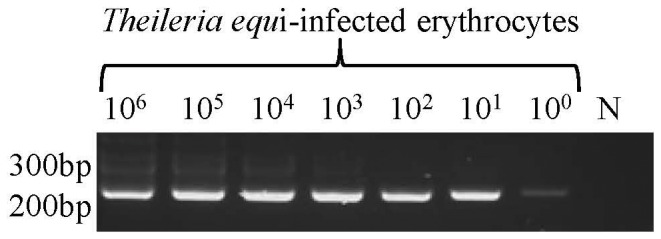
Sensitivity of nested PCR targeting *Theileria equi ema*-1. A 2% agorose gel showing *ema*-1 amplicons from serial 10-fold dilutions of *Theileria equi* infected erythrocytes. N: negative control. The position of the molecular size markers are indicated on the left.

## Materials and Methods

### Ethics Statement

This study was approved by the Institutional Animal Care and Use Protocol Committee of the University of Idaho, Moscow. In addition, we have obtained the owner’s consent for all the horses involved in this study.

**Table 1:** Elimination of *Theileria equi* in the peripheral blood following imidocarb dipropionate treatment.

	Detection of *Theileria equi* in the peripheral blood				
Horse number	Pre-treatment	Post-treatment			
**Treatment 1**	**Day 0**	**Mo.1**	**Mo.3**	**Mo.4**	**Mo.11**
1	2.2×10^3^	nd	nd	nd	nd
84	2.2×10^5^	nd	nd	nd	nd
92	1.2×10^4^	nd	nd	nd	nd
200	2.5×10^5^	nd	nd	nd	nd
300	7.0×10^4^	nd	nd	nd	nd
**Treatment 2**	**Day 0**	**Mo.1**	**Mo.4**	**Mo.8**	**Mo.12**
113	2.5×10^4^	nd	nd	nd	nd
115	2.2×10^4^	nd	nd	nd	nd
118	1.4×10^5^	nd	nd	nd	nd
119	2.7×10^3^	nd	nd	nd	nd
120	5.2×10^4^	nd	nd	nd	nd
121	1.3×10^4^	nd	nd	nd	nd
122	4.7×10^4^	nd	nd	nd	nd
125	1.4×10^3^	nd	nd	nd	nd
126	2.3×10^3^	nd	nd	nd	nd
**Treatment 3**	**Day 0**	**Mo.3**	**Mo.5**	**Mo.8**	**Mo.12**
L1	2.7×10^4^	**Pos**	**Pos**	**Pos**	**Pos**
L2	9.1×10^4^	nd	nd	nd	nd
L4	4.7×10^3^	nd	nd	nd	nd
S2	6.6×10^4^	nd	nd	nd	nd
100	3.3×10^5^	nd	nd	nd	nd
131	1.0×10^5^	nd	nd	nd	nd
153	2.3×10^3^	nd	nd	nd	nd
309	1.5×10^5^	nd	nd	nd	nd
311	4.7×10^4^	nd	nd	nd	nd
319	2.3×10^5^	nd	nd	nd	nd
324	9.2×10^4^	nd	nd	nd	nd

Treatment 1, 2, and 3: distinct horse groups treated at 5 months intervals with the same imidocarb regimen (4.0 mg/kg at 72 h intervals for a total of four injections).

Day 0: levels of *T. equi* per ml of blood prior to treatment by quantitative PCR.

Mo: months post-treatment.

nd: *T. equi* not detectable in the peripheral blood by nested PCR.

Pos: *T. equi* detectable in the peripheral blood by nested PCR.

### Animals and Pathogen

A Quarter Horse mare from Kleberg County, Texas with clinical signs of equine piroplasmosis was diagnosed with *T. equi* infection [20]. Subsequently, all 360 horses on the ranch were serologically tested at the Animal Plant Health Inspection Service, National Veterinary Service Laboratories, U.S. Department of Agriculture, Ames, IA and 292 (81%) were positive for *T. equi* antibodies screened by competitive enzyme-linked immunosorbent assay (VMRD, Inc.). Twenty five of these 292 naturally infected horses were chosen at random for inclusion in this study and were individually identified with the following numbers: L1, L2, L4, S2, 1, 84, 92, 100, 113, 115, 118, 119, 120 121, 122, 125, 126, 131, 153, 200, 300, 309, 311, 319, and 324. The age of the horses included in this study ranged from 2 to 13 years. Blood samples from these infected horses were collected prior to treatment and genomic DNA isolated. Briefly, blood was collected in ethylenediaminetetraacetic acid (EDTA) and washed once with phosphate buffered saline, pH 7.2, to remove the white blood cells. One hundred µl of packed cells were added to 500 µl of RBC lysis solution (QIAGEN, Inc.). The pellets were re-suspended in 450 µl of Cell lysis solution (QIAGEN, Inc.) with 50 µl of proteinase K (2 mg/ml of Cell lysis solution) and incubated at 56°C overnight. Following incubation, 1 µl of glycogen (20 mg/ml) was added to the samples. Then proteins were precipitated with protein precipitation solution (QIAGEN, Inc.). Genomic DNA was recovered in isopropanol, washed in 70% ethanol, and resuspended in 50 µl of DNA hydration solution (QIAGEN, Inc.). All genomic DNA samples were tested for confirmation of *T. equi* infection prior to imidocarb treatment by quantitative PCR and nested PCR as previously described [28].

**Table 2:** Blood transfer following imidocarb dipropionate treatment into naïve horse.

	Pre-blood transfer		Post-blood transfer	
Recipient horse	cELISA	nested PCR	Giemsa-stained blood smears d	nested PCR
Ho-176^a^	–	–	nd	nd
Ho-193^a^	–	–	nd	nd
Ho-178^a^	–	–	nd	nd
Ho-231^a^	–	–	nd	nd
Ho-268^a^	–	–	nd	nd
Ho-194^b^	–	–	Positive	9 days
Ho-228^b^	–	–	Positive	14 days
Ho-267^c^	–	–	Positive	7 days

a recipients for blood transfer from treated horses with nested PCR negative.

b recipients for blood transfer from control horse.

c recipient for blood transfer from treated horse (L1) with nested PCR positive.

d microscopic examination of a minimum of 50 high-power fields.

nd: *T. equi* not detectable in the peripheral blood.

### Imidocarb Dipropionate Treatment of Persistently Infected *T. equi* Horses

All 25 naturally infected horses were treated with imidocarb dipropionate (Imizol®; Schering Plough Animal Health) using a dose of 4.0 mg/kg, intramuscularly, at 72 h intervals for a total of four injections [26]. These horses were divided into three treatment groups and each group was treated at five month intervals to demonstrate reproducibility of the results. A persistently infected horse (horse 98) was left untreated and was used as a positive control for blood transfer transmission studies. Adverse effects associated with the anticholinesterase activity of imidocarb dipropionate as previously described [29] were common in the treated horses. These effects manifested clinically as diarrhea and spasmodic colic, which occurred within 15–20 minutes of injection. The anticholinergic drug N-butylscopolammonium bromide (Buscopan®, Boehringer Ingelheim Animal Health), administered intravenously at 0.3 mg/kg, was effective in ameliorating these adverse cholinergic effects. To lower the probability of exposure to infected ticks, all treated horses were moved to pastures which had never pastured horses and were separated from infected pastures by 0.25 to two miles. An additional buffer zone was added in these pastures with two fence lines and mowed strips of grass in between. All horses were sprayed topically every 14 days with pyrethroid acaricides to prevent tick infestation.

**Table 3:** Percentage of inhibition of specific horse antibodies to *Theileria equi.*

	% of inhibition				
Horse number	Pre-treatment	Post-treatment			
**Treatment 1**	**Day 0**	**Mo.1**	**Mo.3**	**Mo.4**	**Mo. 11**
1	84.59	80.46	60.93	52.46	**27.45**
84	80.05	85.07	50.13	48.67	51.05
92	72.28	75.79	56.87	59.0	43.74
200	87.34	76.96	82.66	86.16	74.40
300	85.35	66.43	48.76	40.66	**18.91**
**Treatment 2**	**Day 0**	**Mo.3**	**Mo.4**	**Mo.8**	**Mo.12**
113	73.36	62.71	64.75	**38.68**	**38.22**
115	70.71	50.63	51.60	**18.91**	**25.99**
118	68.72	52.86	49.90	**19.00**	**26.95**
119	74.40	63.64	58.25	**34.76**	**38.74**
120	76.06	77.71	75.58	59.59	67.50
121	82.13	65.53	63.15	46.08	**38.67**
122	51.18	**27.47**	**29.22**	**12.55**	**17.04**
125	66.48	63.68	64.32	46.46	58.32
126	71.80	68.78	73.68	56.97	64.61
**Treatment 3**	**Day 0**	**Mo.3**	**Mo.5**	**Mo.8**	**Mo.12**
L1	82.97	84.61	84.80	89.14	89.87
L2	79.54	65.02	69.60	55.89	61.84
L4	69.20	58.47	63.82	51.32	48.22
S2	74.21	64.76	66.84	68.34	57.24
100	84.05	71.75	64.74	57.19	64.37
131	84.28	73.85	71.91	65.79	68.73
153	83.71	77.10	75.95	69.88	73.03
309	79.39	68.93	58.79	51.80	41.87
311	79.54	64.66	65.55	54.64	60.37
319	86.68	71.51	71.39	51.68	**34.44**
324	84.91	72.11	62.77	56.96	61.25

Day 0: antibodies inhibition levels indicates *T. equi* infection (pre-treatment).

Mo: months post-treatment.

Percentage of inhibition in cELISA <40% indicates reverted to seronegative status (indicated by bold number).

### Detection of *T. equi* Elimination and Serological Status of Treated Horses

Prior to treatment and at multiple time-points following treatment, the presence of the parasite in peripheral whole blood was tested by nested PCR and level of parasites was quantified by real-time PCR targeting the *T. equi* single copy *ema-*1 gene as previously described [14]. To determine the sensitivity of nested PCR, infected blood from a splenectomized horse with high parasitemia (Giemsa stained blood smears: 1.4×10^8^ infected erythrocytes/ml of blood) was collected and genomic DNA extracted. Real time PCR determined that the level of organisms was 4.2×10^8^ *T. equi*/ml of blood. Then 10 fold dilutions (10^6^−10^0^ infected erythrocytes), followed by genomic DNA isolation as described above and nested PCR, were performed to determine the sensitivity. The sensitivity of nested PCR was determined to be <10 parasites (Fig. 1). A previous publication demonstrated similar sensitivity results in which nested PCR was capable of detecting six infected erythrocytes corresponding to parasitemia of 6×10^−6^% [30]. The sensitivity of quantitative real-time PCR was determined to be between 10 to 100 parasites as previously described [28]. The official regulatory cELISA was used to detect *T. equi* specific serum antibodies. Treated horses with antibody levels resulting in <40% inhibition in the cELISA were considered seronegative (VMRD, Inc.).

### Blood Transfer to Determine *T. equi* Transmission Risk in Imidocarb Dipropionate Treated Horses

From each of the horses treated with imidocarb dipropionate, 500 ml whole blood was collected in citrate phosphate dextrose anticoagulant (Fenwal Inc.) at 4–5 months post-treatment and shipped on ice (cooler packs) overnight to the U.S. Department of Agriculture-Agricultural Research Service-Animal Disease Research Unit facility in Pullman, WA for subsequent intravenous inoculation into a naïve splenectomized horse [26]. Blood was shipped for this purpose on three separate occasions. To minimize the number of recipient horses, 120 ml blood from each of 4–5 treated horses was inoculated separately into a single recipient upon arrival. Recipient Ho-193 received a total of 600 ml whole blood from treated horses 1, 84, 92, 200 and 300; Ho-178 received 600 ml whole blood from treated horses 113, 115, 118, 119 and 120; Ho-231 received 480 ml whole blood from treated horses 121, 122, 125 and 126; Ho-268 received 600 ml whole blood from treated horses L2, L4, 52, 100 and 131; and Ho-176 received 600 ml whole blood from treated horses 153, 309, 311, 319 and 324. As a positive control for the first two shipments of blood, recipient horses Ho-194 and Ho-228 each received 120 ml of blood from untreated infected horse 98. Finally, as a positive control for the third shipment, recipient horse Ho-267 received 120 ml of blood from infected horse L1 that was treated with imidocarb dipropionate but remained infected as determined by nested PCR. All recipient horses were monitored daily for clinical signs of piroplasmosis for approximately 60 days post-blood transfer. Blood samples were collected weekly to determine *T. equi* infection status by microscopic examination of Giemsa stained blood smears and by nested PCR.

### Statistical Analysis

A Fisher’s exact test was performed using SAS 9.2 software (SAS Institute, Cary, NC) to determine the two-tailed p value and exact 95% confidence interval for the proportion of successfully treated horses (those in which peripheral blood elimination of *T. equi* occurred as determined above).

## Results

### Imidocarb Dipropionate Treatment of Persistently Infected *T. equi* Horses

Prior to imidocarb dipropionate treatment, all 25 naturally infected horses had levels of *T. equi* parasitemia consistent with the persistent phase of infection [17], ranging from 1.4×10^3^ to 2.2×10^5^ parasites per ml of peripheral blood (Table 1). After imidocarb dipropionate treatment, *T. equi* was no longer detectable in peripheral blood by either quantitative real-time PCR or nested PCR assays in 24 of the treated horses. These horses remained nested PCR negative through 12 months of follow-up (Table 1). In contrast, imidocarb dipropionate failed to eliminate *T. equi* in horse L1, which remained PCR positive with levels of parasitemia ranging from 1.9×10^5^ to 4.3×10^6^ *T. equi* per ml of blood.

### Transfer of Blood from Treated Horses into Naive Recipient Horses

With the exception of horse L1, transfer of blood from treated horses into naive splenectomized horses did not transmit *T. equi* to the recipients, as determined by lack of clinical disease and failure to detect parasites in peripheral blood on Giemsa stained blood smears or by nested PCR throughout 60 days of follow-up (Table 2). In contrast, blood transfers from untreated PCR positive control horse 98, and unsuccessfully treated horse L1, readily transmitted *T. equi* to naïve recipient splenectomized horses, which developed signs of equine piroplasmosis between 7 to 14 days post blood transfer (Table 2). Overall, imidocarb dipropionate treatment resulted in peripheral blood *T. equi* elimination in 24 of 25 horses (96%). This level of therapeutic efficacy was highly significant, with a Fisher’s exact test 95% CI range of 79.7–99.9% (p<0.0001). Importantly, detection of *T. equi* by nested PCR was 100% concordant with the results of blood transfer transmission to naïve splenectomized recipients, suggesting that nested PCR could be used to predict peripheral blood elimination of *T. equi* in naturally infected horses.

To determine if the *T. equi* subset population in horse L1 was resistant to imidocarb dipropionate, at 12 months after the first round of treatment, a second round of treatment was performed which eliminated infection as determined by nested PCR and confirmed by blood transfer into a naïve splenectomized horse. The recipient horse showed no clinical signs of equine piroplasmosis and nested PCR was negative throughout 60 days of follow-up (data not shown).

### *T. equi* Antibody Levels following Peripheral Blood Elimination of Parasites

Despite the therapeutic efficacy of imidocarb dipropionate for *T. equi* peripheral blood elimination as determined above, EMA-1-specific serum antibody levels as measured by cELISA were slow to decline following treatment, persisting throughout the 12 month follow-up period in some horses. For the first treatment group, levels of *T. equi*-specific serum antibodies declined three months post-treatment in four horses, but remained unchanged in one horse (Table 3). At 11 months post-treatment, two horses became seronegative, while three remained seropositive (Table 3).

For the second treatment group, serum antibody levels at three months post treatment declined in five horses and one horse became seronegative (horse 122). No decline in *T. equi*-specific serum antibody levels was observed at three months post-treatment in the other three horses in this group. In addition to horse 122, four other horses in this group became seronegative at eight months post-treatment (horses 113, 115, 118 and 119), while four horses remained seropositive. At twelve months post-treatment, the five seronegative horses remained seronegative and one other horse became seronegative while the three seropositive remained seropositive (Table3).

For the third treatment group, serum antibody declined at three months post treatment in ten horses with a further slight decline at eight months post-treatment (Table 3). At twelve months post treatment, one horse (horse 319) became seronegative while the nine other horses remained seropositive (Table 3). In contrast, horse L1, which remained consistently PCR positive following the first imidocarb dipropionate treatment, showed no changes in the levels of *T. equi* antibodies throughout the 12 months of follow-up (Table 3).

## Discussion

Naturally infected *T. equi* carrier horses without overt signs of infection represent a source of pathogen for iatrogenic and/or tick transmission to susceptible horses [14], [17]. Chemotherapeutic elimination of *T. equi* from persistently infected horses represents a critical component of a control strategy for preventing the spread of this pathogen in non-endemic areas such as the United States. However, the achievement of chemotherapeutic elimination and the subsequent confirmation thereof can be problematic when treating infectious diseases caused by persistent parasites. In this study, we demonstrated that imidocarb dipropionate was efficacious for the elimination of *T. equi* from the peripheral blood of naturally infected carrier horses. This conclusion is supported by two lines of evidence: i) the inability to detect *T. equi* organisms in the peripheral blood by nested PCR at multiple time points following imidocarb dipropionate treatment, and ii) the inability of transfused whole blood from nested PCR negative treated horses to infect highly susceptible naïve splenectomized horses. In contrast, *T. equi* organisms were consistently detected in the peripheral blood of an untreated persistently infected horse, and blood transfer from this horse was able to establish infection in naïve splenectomized horses.

We hypothesized that chemotherapeutic *T. equi* elimination would result in declining levels of *T. equi*-specific serum antibodies, and that treated horses would become seronegative within several months [26]. Although nested PCR failed to detect *T. equi* in successfully treated horses throughout the study, the official regulatory cELISA diagnostic test indicated that only a few treated horses became seronegative, with the majority of horses remaining seropositive up to 12 months post-treatment. The possible explanations of long-term antibody persistence in these nested PCR negative treated horses include: i ) a low level of *T. equi* organisms not detectible by nested PCR stimulating an immune response; and ii) long lived plasma cells secreting *T. equi*-specific antibodies. Low levels of *T. equi* in the peripheral blood were excluded by testing treated horses at multiple time points using nested PCR. Studies with *Babesia bovis*, a closely related pathogen of cattle, indicate that levels of parasitemia fluctuate during chronic infection, and that testing peripheral blood at multiple time-points using PCR readily detects parasites [31]. In contrast, transfer of large volumes of whole blood into naïve splenectomized horses in the current study failed to reveal low levels of parasitemia, consistent with our negative nested PCR results. Taken together, these results indicated that *T. equi* was eliminated by imidocarb dipropionate. The most likely explanation for long-term *T. equi*-specific antibody persistence was the maintenance of long-lived plasma cells secreting antibodies in the absence of specific antigens. Long-term antibody persistence in other infectious disease models following elimination of infection has been reported [32], [33].

The extended time between chemotherapeutic elimination of *T. equi* and the first seronegative result could be problematic, as horses that no longer pose a transmission risk would remain under prolonged quarantine according to current regulations. Based on this potential challenge, our laboratory is focused on identifying an antigen against which post-elimination antibodies decline more rapidly than the Equi Merozoite Antigens 1 and 2 (EMA1 and EMA2)-specific antibodies detected in the currently licensed cELISA. The goal is to identify antigens that could serve as potential immunologic markers for the development of a rapid and accurate serologic assay to confirm chemotherapeutic elimination of *T. equi*.

An important observation in this study is that imidocarb dipropionate treatment failed to eliminate *T. equi* from the peripheral blood of one of the 25 treated horses. In this horse, *T. equi* organisms persisted after treatment as determined by positive nested PCR at multiple time points, and parasite viability was demonstrated by blood transfer into a naïve splenectomized horse. Interestingly, the second treatment of horse L1 eliminated infection indicating that this *T. equi* subset population was not resistant to imidocarb dipropionate. The failure of one course of imidocarb treatment to eliminate *T. equi* infection in one horse could be due to individual differences in drug metabolism or drug distribution. Alternatively, the relapsing of parasites in the peripheral blood following one course of treatment may have been caused by the failure of imidocarb to completely eliminate the pre-erythrocytic stages of *T. equi* in this horse infected through natural tick-borne transmission. Previous studies have demonstrated that *T. equi* sporozoites infect peripheral blood mononuclear cells and then develop into schizonts prior to the infection of erythrocytes [16]. In a closely related human pathogen, *Plasmodium vivax*, sporozoites infect liver cells prior to infecting erythrocytes. Anti-malaria drugs such as chloroquine have failed to eliminate *P. vivax* liver stages [34]. These liver stages can develop into schizonts which are then released as merozoites into the peripheral blood causing relapsing malaria [34]. Whether a similar mechanism during the initial *T. equi* mononuclear cell infection contributed to the initial treatment failure in the horse of the current study is unknown. Due to the limited number of anti-protozoal drugs available with efficacy against *T. equi*, horses that remained infected following one course of treatment with imidocarb dipropionate pose a significant transmission risk [22], [35].

In conclusion, the results of this study provide an alternative strategy to control and potentially eradicate equine piroplasmosis in non-endemic areas. In addition to improving animal health, the chemotherapeutic elimination of *T. equi* from persistently infected horses removes the risk of transmission. However, there is a risk that some horses will remain infected after imidocarb treatment. These infected horses represent reservoirs that could maintain the transmission cycle. The ability of imidocarb dipropionate to eliminate *T. equi* infection from persistently infected horses provides a feasible strategy to control sporadic outbreaks and will greatly benefit the international equine trade industry by removing *T. equi* infection as a global restriction on equine movement.
